# First Detection of Human- and Dog-Associated *Demodex* Mites (Acari, Arachnida) in Southern European Wolves (*Canis lupus*)

**DOI:** 10.3390/pathogens15030336

**Published:** 2026-03-21

**Authors:** Natalia Sastre, Manena Fayos, Luca Rossi, Olga Francino, Roser Velarde, Sebastian E. Ramos-Onsins, Lluís Ferrer

**Affiliations:** 1 Servei Veterinari de Genètica Molecular, Departament de Ciència Animal i dels Aliments, Facultat de Veterinària, Universitat Autònoma de Barcelona, 08193 Bellaterra, Spain; 2 Centro de Recuperación de Fauna Silvestre de Cantabria, 39690 Obregón, Spain; 3 Dipartimento di Scienze Veterinarie, Campus di Agraria e Medicina Veterinaria, Università di Torino, 10124 Torino, Italy; 4 Departament de Medicina i Cirurgia Animals, Universitat Autònoma de Barcelona, 08193 Bellaterra, Spain; 5 Centre for Research in Agricultural Genomics (CRAG), CSIC-IRTA-UAB-UB, 08193 Bellaterra, Spain

**Keywords:** *Demodex brevis*, *Demodex canis*, *Demodex folliculorum*, *Demodex injai*, *qPCR*, wolf, *16S rRNA* gene, *18S rRNA* gene

## Abstract

*Demodex* mites are common commensals of mammalian skin, but under certain conditions, they can cause severe skin diseases. This study analyzed the presence, diversity, and phylogenetic relationships of *Demodex* species in two wolf subspecies from southern Europe to determine whether species-level differences exist between wild and domestic canids after thousands of years of divergence. A total of 1400 hair samples from 140 wolves were analyzed using a real-time PCR (qPCR) targeting mitochondrial *16S rRNA* and nuclear *18S rRNA* genes. Overall, 37.1% (52/140; 95% CI: 29.0–45.9%) of wolves were positive for *Demodex* DNA, with a higher prevalence in Italian (46%) than in Iberian (36%) wolves. The lip and chin areas were the most reliable sampling sites. Four *Demodex* species were identified in wolves: *D. injai* and *D. canis* (associated with dogs), and *D. folliculorum* and *D. brevis* (associated with humans). Co-infestations involving multiple *Demodex* species were recorded for the first time in wild canids. These results challenge the long-held belief of strict host specificity in *Demodex* mites. The discovery of *Demodex* species associated with both humans and dogs in wolves supports the idea that host-switching and ecological interactions have occurred throughout the evolution of canids and humans. Such cross-species transfers may have taken place during the early domestication of dogs, representing a plausible scenario compatible with our data. However, given the isolated history of the two southern wolf populations, it is more probable that these findings result from recent interspecific transmission events, likely facilitated by ecological overlap with domestic animals and human environments. Future genomic studies will be essential for clarifying the evolutionary relationships within the genus *Demodex* and its host associations.

## 1. Introduction

*Demodex* mites are considered normal inhabitants in mammalian skin [1,2,3]. Currently, more than 80 species of *Demodex* spp. have been described, genetically and/or morphologically, in more than 80 wild and domestic mammals, including humans, dogs, cats, deer, ferrets, otters, and mice [2,4,5,6,7,8,9,10,11,12,13]. Most mammals harbor *Demodex* mites on the skin without developing lesions or any other clinical signs [1,13]. Multiple pieces of evidence and studies indicate that the immune system controls *Demodex* populations on the skin [1,3,14]. Although *Demodex* mites are considered commensals in small numbers, when they proliferate, they may cause severe dermatitis, for instance, rosacea in humans or demodicosis in dogs and cats [15,16,17]. In almost all cases, proliferation results from a compromised immune system, as occurs in transgenic mice [14,18] or in dogs or humans receiving immunosuppressive medical treatment [3,19,20].

Genetic sequencing has proven highly effective for detecting and identifying *Demodex* mites in both healthy and diseased mammals [4,7,9,13]. Because of the sensitivity and specificity of genetic techniques, mites can be detected where non-molecular methods, such as microscopy or trichoscopy, fail to yield results [21,22]. *Demodex* mites have been detected, identified, and classified in humans, dogs, cats, mice, and ferrets using different genetic approaches such as the mitochondrial *16S rRNA* gene region [4,6,7,9], and the nuclear *18S rRNA* gene region [2,9,23,24]. Ravera et al. (2013) [13], using a fragment of the *chitin synthase* gene, concluded that *Demodex* DNA can be detected in the skin of any dog if 20 or more skin sites are sampled. In wolves, *Demodex* mites have been detected in fecal samples [25,26,27]. Since *Demodex* spp. are hair follicle mites, their presence in feces could be explained by accidental ingestion during grooming or from the prey itself. Nevertheless, it remains unknown which *Demodex* species are present in wolves’ skin.

Research on *Demodex* mites is relevant not only to medicine but also to evolutionary biology. Their parasitic association with mammals may date back approximately 220 million years, coinciding with the emergence of hair follicles [8,28]. Roughly 100 million years ago, the clade Boreoeutheria diverged into two sister lineages: Euarchontoglires, which includes primates, and Laurasiatheria, which includes canids [29]. *Demodex* mites have been documented, both morphologically and genetically, in species from both groups, suggesting either a long-term stability of the hair follicle niche or a high capacity for mite transmission across and within placental mammals [4,5,7,9,24,30,31]. The genus *Demodex* therefore represents an exceptional case of parasitic parallelism [2,8,13,32].

The domestic dog originated from the gray wolf (*Canis lupus*) and is associated with Neolithic humans on most continents [33,34,35]. Axelsson et al. (2012) [36] suggest that dogs descended from scavenger wolves adapted to a starch-rich diet during the dawn of the agricultural revolution. However, it remains unclear where domestication occurred and whether it occurred once or multiple times worldwide. Archaeological records suggest different origins and domestication times: fossilized early dog remains have been found in Belgium (36,000 years ago; [37]), eastern Siberia (33,500 years ago; [38]), and northern Israel (12,000 years ago; [39]). Nevertheless, specimens older than 14,000 years do not appear related to modern dogs [38]. Genetic studies also yield different results. Autosomal single nucleotide polymorphism (SNP) data indicate contributions from Middle Eastern and European wolves [40], while shotgun sequencing data suggest an origin in Eurasia, possibly southern Asia [41]. Wang et al. (2015) [42] propose that domestication began around 33,000 years ago in East Asia, followed by migrations toward the Middle East and Europe. More recently, Bergström et al. (2022) [43] found that modern dogs are genetically closer to ancient wolves from eastern Eurasia than to those from western Eurasia. However, Freedman et al. (2014) [44] found that no extant wolf lineage is more closely related to dogs, implying that an extinct wolf lineage was their ancestor. In any case, dog domestication can be traced back at least 15,000 years.

The gray wolf (*Canis lupus*) is listed on the IUCN Red List (2018; https://www.iucnredlist.org/species/3746/247624660, accessed on 9 February 2026) as “Least Concern” and is included in Appendix II of the Convention on International Trade in Endangered Species of Wild Fauna and Flora (CITES) (except for the populations of Bhutan, India, Nepal, and Pakistan, which are listed in Appendix I). Currently, five subspecies of *Canis lupus* are recognized in North America and seven in Eurasia. This classification is primarily based on genetic and morphological differences among populations, reflecting adaptations to distinct climates, latitudes, or habitats [45,46,47,48]. In Western Europe, the differentiation between the subspecies *C. l. signatus* of the Iberian Peninsula and *C. l. italicus* of the Italian Peninsula is the result of prolonged geographic isolation, which continues to this day [49,50,51,52].

Our study aims to determine whether there are species-level differences in *Demodex* mites between wild and domestic canids after this extensive period of divergence. For this purpose, we analyzed 1400 hair samples from 140 wolves belonging to the two southern European subspecies, using two gene fragments, 16S rDNA and 18S rDNA, widely used for the molecular identification of *Demodex* mites. We specifically aim to identify the *Demodex* species present in wolves, compare them with those found in domestic dogs, and assess whether host domestication and divergence may have influenced mite diversity. By addressing these questions, we test the hypothesis that long-term host divergence and domestication may have resulted in distinct *Demodex* species in wild and domestic canids, providing new insights into the evolutionary history of these mites.

## 2. Materials and Methods

### 2.1. Ethics Statement

In Cantabria, wolves were classified as a hunting species under Annex I of the Cantabrian Law, 12/2006. From 2017 to 2020, wolves were removed under a species-control program implemented by “Subdirección General del Medio Natural” of the Government of Cantabria, in accordance with Laws 2/2017, 5/2018, and MED/5/2019, under expedient numbers CVE-2017-1827, CVE-2018-17138, and CVE-2019-2950, to reduce wolf attacks on livestock in conflict areas. From 2021 to 2025, wolves in the Iberian Peninsula were protected, and hunting was banned. Individuals necropsied from 2021 onwards died accidentally. Natural Environment agents transported the carcasses to the Wildlife Rescue Center of Cantabria for necropsies. The three wolves from Aragón, Castilla y León and Catalunya died after being hit by a car. Hair samples were taken by agents of the Natural Environment for necropsies. Individuals from Italy (where wolf hunting is banned) died accidentally. Technicians from the European project Life12 NAT/IT/000807 WolfAlps moved carcasses to the Department of Veterinary Science (Università degli Studi di Torino) for necropsies. All procedures involving animal carcasses complied with national and regional regulations and did not involve the deliberate killing of animals for research purposes.

### 2.2. Sampling

Hair samples from Iberian and Italian wolves were obtained from northern Spain, including Cantabria (126 samples), Aragón (1), Castilla y León (1), and Catalunya (1), and from the Piemonte region of northern Italy (11) (Table 1). One hundred twenty-seven wolves (61 females and 66 males) from Cantabria and Castilla y León were morphologically and genetically identified as *Canis lupus signatus*, and 13 wolves (6 females and 7 males) from Piemonte, Catalunya, and Aragón were identified as *Canis lupus italicus* (Table 1). Between 2017 and 2025, we collected a total of 1400 hair samples from 140 wolf carcasses, with 10 samples per carcass obtained during necropsy examinations (Table 1 and Table S1). Five sites were from the face: periocular, lips, nose, chin, and entrance to the external ear canal, and five sites were from the rest of the body: dorsum, lumbar, abdomen, forelimb, and hindlimb. Using gloved hands and surgical mosquito forceps, hair was plucked in the direction of growth to include the hair bulb (root) for DNA extraction. Hair samples were stored at −20 °C until DNA extraction.

### 2.3. DNA Extraction and Real-Time PCR (qPCR) Amplification

Over the years, DNA from hair bulbs has been extracted using two different methods (Table S1): Tris-HCl approach, following Ravera et al. 2013 [13]; and PerkinElmer automated extraction, Chemagic 360 Instrument (Baesweiler, Germany), following the manufacturer’s protocol, including an incubation step at 56 °C overnight and a final elution volume of 50 µL DNA. An extraction blank was included in each 96-well plate. All extracted DNA was diluted 1:10 for qPCR amplification.

16S and 18S primers were used to amplify approximately 300-bp and 500-bp fragments of the mitochondrial *16S rRNA* gene and the nuclear *18S rRNA* gene, respectively [9]. All DNA samples were amplified by real-time qPCR and prepared under a laminar flow hood. Positive qPCR controls were obtained from known *Demodex* mite DNA [9]. Duplicates were amplified for each sample, and two extraction blanks and one negative qPCR control per gene were included in each 384-well plate to detect any qPCR exogenous DNA contamination. qPCR amplifications were performed in a QuantStudioTM 12K Flex Real-Time PCR System (Thermo Fisher Scientific, Waltham, MA, USA). Amplicons were sequenced and purified when melting curves showed Tm = 75 ± 1 °C for the *16S rRNA* gene and Tm = 83 ± 2 °C for the *18S rRNA* gene, and amplification cycles had Cp < 37. Sequences were separated on an ABI PRISM 3730 automated sequencer (Thermo Fisher Scientific) according to the manufacturer’s instructions.

### 2.4. Genetic Variability and Phylogenetic Analysis

Genetic diversity in *Demodex* mites was analyzed using DNASP 5.10 [53]. For comparisons and phylogenetic analysis, we used mite sequences from wolves (those ending in UAB) and those available in GenBank. Sequences were analyzed using SEQSCAPE 2.1.1 software (Thermo Fisher Scientific) and were compared with the GenBank database (www.ncbi.nlm.nih.gov/BLAST, accessed on 1 May 2025). Phylogenetic analysis for the mitochondrial *16S rRNA* gene was carried out using 44 sequences and 302 bp (gaps included). Phylogenetic analysis for the *18S rRNA* gene was carried out using 45 sequences and 491 bp (gaps included). The trees were rooted using outgroups from the class Pycnogonida, *Achelia hispida* (FJ862845) and *Ammothea* sp. (FJ862841) for the *16S rRNA* gene, and *Achelia echinata* (AF005438) and *Callipallene* sp. (AF005439) for the *18S rRNA* gene. MODELTEST 3.7 [54] was applied to select the best evolutionary model among 56 models of evolution by the Akaike information criterion. The Bayesian program MrBayes v3.2.7 [55] was implemented to generate the phylogenetic tree with 1,000,000 Markov Chain Monte Carlo iterations and a burn-in rate of 25%. Bootstrap values indicate the repeatability of the inferred clades and may provide a conservative estimate of their accuracy [56]. To display the phylogenies, we used the program FigTree (http://tree.bio.ed.ac.uk/software/figtree/, accessed on 1 September 2025).

## 3. Results

### 3.1. Mite Prevalence in Wolves

We screened 1400 hair samples from 140 wild wolves for mite DNA targeting the *16S rRNA* and *18S rRNA* genes. qPCR samples were considered positive when the melting curves (Tm) were close to *Demodex* control values (Tm = 75 °C ± 0.5 °C, 16S rDNA; Tm = 81.5 °C ± 0.5 °C, 18S rDNA), and at least one gene was successfully sequenced. The prevalence of *Demodex* mites in the hairs of 140 wolves was 37% (52/140; 95% CI: 29.0–45.9%). Of the Italian wolves, 46% (6/13; 95% CI: 19.2–73.9%) were positive for *Demodex*, while 36% were positive among Iberian wolves (46/127; 95% CI: 27.8–45.4%) (Table 1 and Table S1). Given the small sample size of Italian wolves, this prevalence should be interpreted with caution. No bias toward females and males (χ^2^ (1, N = 140) = 1.02, *p* > 0.05) was observed among *Demodex*-positive wolves.

Sampling 10 sites, 101 out of 1400 hair samples tested positive for *Demodex* by qPCR (Table S1). Twenty-three wolves (44%) were positive at one site, nineteen wolves (36%) at two sites, six wolves (12%) at three sites, two wolves (4%) at four sites, and two wolves (2%) at five and nine sites, respectively. *Demodex* positivity was significantly higher in face samples (71%) compared to body samples (χ^2^ (1, N = 1400) = 19.73, *p* < 0.001). The most likely site to detect *Demodex* mites was the lip area (20%), followed by the chin area (18%). The least common sites were the lumbar (4%) and the dorsal (3%) areas.

Regarding DNA extraction methods, the PerkinElmer automated extraction was significantly more efficient than Tris-HCl approach (χ^2^ (1, N = 1400) = 22.56, *p* < 0.0001), with 85/869 positives (10%) versus 16/531 (3%). Although the distribution of extraction methods differed between Italian (53% Perkin; N = 130) and Iberian (63% Perkin; N = 1270) wolves (χ^2^ = 4.87, *p* = 0.027), this did not affect the comparison of positive detection rates. A Cochran–Mantel–Haenszel test adjusting for both species confirmed a significant association between extraction method and positivity (common OR = 3.48, 95% CI: 2.02–6.00; χ^2^(1) = 22.7, *p* < 0.0001). However, the Breslow–Day test for homogeneity of odds ratios was not significant (χ^2^(1) = 0.94, *p* = 0.33), indicating that the Perkin method’s superior efficacy was consistent across wolf subspecies. A significant disadvantage of the Tris-HCL method is that DNA is not purified, reducing qPCR success due to higher inhibitor levels, which may lead to false-negative results. Its main advantages are its speed and low cost.

### 3.2. Identification of Demodex spp.

Using the *18S rRNA* gene, 83 samples were positive for *Demodex* spp., 21 samples could not be identified due to different mite types in the same sample (double sequence), and 88 samples were positive for other acarid mites (Table S1). Among the positive *Demodex* samples, we identified *Demodex injai* (52), *Demodex canis* (26), and *Demodex folliculorum* (5), but not *Demodex brevis* (Table 2 and Table S1). Using the *16S rRNA* gene, we identified 101 positive samples, including *D. injai* (54), *D. canis* (30), *D. folliculorum* (12), and *D. brevis* (5) (Table 2 and Table S1).

Interestingly, the *Demodex* species commonly reported in dogs, such as *D. canis* and *D. injai*, were not detected in any of the Italian wolves examined. Instead, two species commonly associated with humans, *D. folliculorum* and *D. brevis*, were identified. Differential sampling effort (0 positives in 13 Italian wolves versus 45 positives in 127 Iberian wolves) could potentially bias detection probabilities, even though it was statistically significant (Fisher’s exact test, *p* = 0.015). A larger sample size would be necessary to determine whether *D. canis* and *D. injai* are absent from this wolf subspecies, especially considering that a recent study found evidence of extensive wolf–dog hybridization in peninsular Italy [57]. In contrast, all four *Demodex* species, those usually reported in both dogs and humans, were identified in Iberian wolves. *D. injai* showed the highest prevalence, present in more than half of the individuals (64%), followed by *D. canis* (24%), while *D. brevis* and *D. folliculorum* had lower rates (8% and 4%, respectively). Additionally, co-infestation with *D. canis* and *D. injai* and *D. canis* and *D. brevis* were observed in three and one Iberian wolves, respectively, and *D. brevis* and *D. folliculorum* in one Italian wolf (Table 2 and Table S1).

### 3.3. 16S rDNA Sequence Variability and Phylogenetic Relationships

We identified 11 *Demodex* sequences (haplotypes) among 101 sequences of the mitochondrial *16S rRNA* gene from 52 wolves (Table 2 and Table S1). The 11 *Demodex* haplotypes were submitted to GenBank because they had not been previously described in wolves and, in most cases, represented new records (Table 2).

They were identified as follows: (1) *Demodex injai* (three haplotypes): D.injai_UABW1 (N = 19 sequences, identical to the sequence KT259449 from GenBank), D.injai_UABW2 (N = 29 sequences), and D.injai_UABW3 (N = 6 sequences); (2) *Demodex canis* (two haplotypes): D.canis_UABW1 (variant *cornei*) (N = 27 sequences, identical to sequences JX390979 and MN161404 from GenBank), and D.canis_UABW2 (N = 3 sequences, identical to several sequences from GenBank such as JF784000); (3) *Demodex folliculorum* (three haplotypes): D.folliculorum_UABW1 (N = 7 sequences, identical to sequences HQ844221, FN424245, and FN424246 from GenBank), D.folliculorum_UABW2 (N = 3 sequences), and D.folliculorum_UABW3 (N = 2 sequences); and (4) *Demodex brevis* (three haplotypes): D.brevis_UABW1 (N = 2 sequences), D.brevis_UABW2 (N = 2 sequences), and D.brevis_UABW3 (N = 1 sequence). Haplotypes from *D. injai* and *D. canis* were exclusive to the Iberian wolves, as well as haplotypes D.folliculorum_UABW3, D.brevis_UABW1, and D.brevis_UABW3 (Table 2). The exclusive haplotype found in Italian wolves was D.folliculorum_UABW2. Finally, haplotypes D.folliculorum_UABW1 and D.brevis_UABW2 were shared between wolf populations.

**Figure 1:**
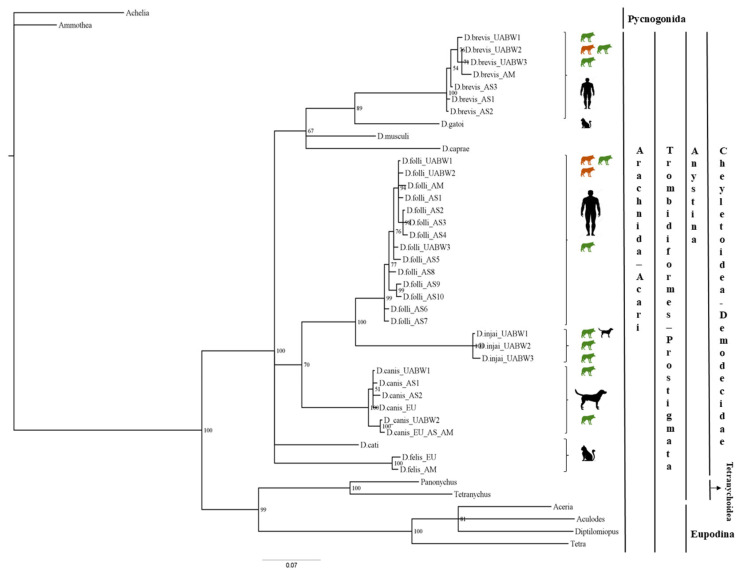
Phylogenetic analyses of *Demodex* spp. The tree was estimated using MrBayes based on aligned fragments of the *16S rRNA* gene. Branch support is based on 10,000 bootstrap replications. Bootstrap values are shown as percentages. The scale at the bottom represents genetic distances, expressed as the number of nucleotide substitutions per site. Green: *Canis l. signatus*; Brown: *Canis l. italicus*.

We conducted a phylogenetic analysis that included the 11 *Demodex* sequences, 31 sequences from the order Trombidiformes, and two outgroups obtained from GenBank (Figure 1). In total, we aligned 44 fragments of 302 bp each (gaps included). The Tamura-Nei substitution model, which assumes equal base frequencies and accounts for among-site rate heterogeneity using a gamma distribution (TrNef+G) was identified as the best-fit model for the *16S rRNA* gene.

The 16S-Bayesian tree (Figure 1) showed two major splits among the Trombidiformes order (bootstrap value 100%). All our samples belong to the Anystina cohort, which includes the family Demodecidae. However, the Demodecidae sequences did not cluster by host species. For instance, *D. brevis* (host: human) was sister taxa with *D. gatoi* (host: cat), and *D. folliculorum* clade (host: human) was sister to the *D. injai* clade (host: dog) with strong bootstrap support (100%). Despite the short length of the 16S fragment, which limits deeper evolutionary inference, these results suggest that the evolutionary relationships among *Demodex* mites may not strictly follow host phylogeny, indicating possible host-switching events over evolutionary time.

### 3.4. 18S rDNA Sequence Variability and Phylogenetic Relationship

We identified three *Demodex* sequences (haplotypes) among 83 sequences of the nuclear *18S rRNA* gene from 40 wolves (Table 2 and Table S1). The three *Demodex* haplotypes were submitted to GenBank because they had not been previously described in wolves (Table 2). They were identified as *Demodex injai*, *Demodex canis* and *Demodex folliculorum*, and they have been described previously in dogs and humans (Table S1).

In addition to *Demodex*-positive samples, we sequenced 49 samples that amplified at Cp < 37 and melting curves Tm = 82 °C + 3 °C (Table S1). We amplified DNA from 11 mites belonging to the order Oribatida, 3 to the order Prostigmata, and one to the order Ixodida, the only hematophagous mite detected in an Iberian wolf (Table S1). The remaining mites were most likely soil-dwelling and not host-associated. Table S1 shows the sequences of the 15 mites, along with their percentage identity relative to the reference sequence in GenBank. The genus names used in the phylogenetic tree should be considered tentative, as the order-level classification is reliable, but species-level identification is not possible with the available data. These findings indicate that the 18S rDNA fragment is not an optimal marker for detecting *Demodex* in wolves.

**Figure 2:**
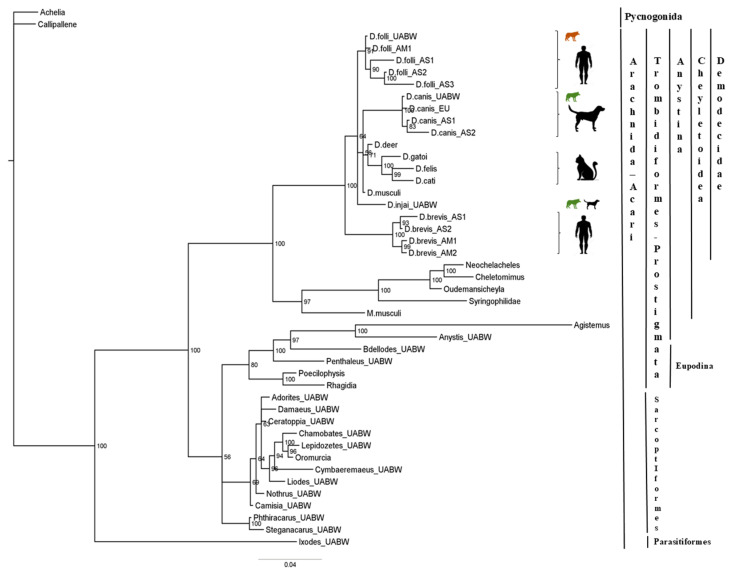
Phylogenetic analyses of *Demodex* spp. The tree was estimated using MrBayes based on aligned fragments of the *18S rRNA* gene. Branch support is based on 10,000 bootstrap replications. Bootstrap values are shown as percentages. The scale at the bottom represents genetic distances, expressed as the number of nucleotide substitutions per site. Green: *Canis l. signatus*; Brown: *Canis l. italicus*.

The 18S-Bayesian tree (Figure 2) showed a clear split (bootstrap value 100%) between the Parasitiformes (Ixodida) and the Sarcoptiformes (Oribatida) and the Trombidiformes (Prostigmata) Orders. However, Oribatida did not form a distinct cluster, whereas Prostigmata split into several clusters, including the Eupodina and Anystina cohorts. The Demodecidae family constitutes a monophyletic group of the Anystina cohort that includes four large clusters: (1) the *D. folliculorum* cluster (host: human), (2) the *D. canis* cluster (host: dog), (3) the *D. gatoi*, *D. cati*, and an unnamed *Demodex* species in a cat (here referred to as *D. felis*) cluster (host: cat), and (4) the *D. brevis* cluster (host: human). Again, *D. injai* appears to be more closely related to *D. folliculorum* than to *D. canis*; however, this result should be interpreted with caution due to the low bootstrap support (<70%).

## 4. Discussion

### 4.1. Detection, Prevalence, and Identification of Demodex spp.

*Demodex* mites were detected in over one-third of the wolves, with a slightly higher prevalence in Italian than in Iberian wolves and no sex bias, suggesting widespread infestation in both populations. Detection of *Demodex* DNA, however, does not necessarily reflect an active infestation or viable mites. Furthermore, it is important to emphasize that the wolves in this study were wild. In a previous study, in which we examined five body regions from 14 Mexican (*Canis lupus baileyi*) and 8 Alaskan (*Canis lupus occidentalis*) captive wolves, we were unable to detect any positive cases with confidence [9]. The fact that these captive animals were vaccinated annually, fed regularly, and kept in sheltered conditions possibly reduced mite proliferation and, consequently, the likelihood of detection, despite our assumption that all wolves harbor *Demodex* mites in their pilosebaceous complexes. Notably, to detect 37% of wolves positive for *Demodex*, each wolf had to be sampled at 10 different skin sites, yielding a total of 1400 samples, of which only 7% tested positive. Therefore, it is more effective to examine fewer wolves across multiple sites than to explore a larger number of wolves at a single site [1,9,13]. Based on our results, the number of sampling sites for detecting *Demodex* mites can be limited to the face, with the most suitable sites being the lips and chin. The dorsal and lumbar regions can be excluded. Reducing the number of sites decreases laboratory workload and overall costs. The resulting time and resources are recommended for use in a DNA extraction method incorporating a purification step to minimize the risk of false negatives, despite its higher cost relative to non-purified DNA extraction.

Regarding the genetic markers, our results suggest that the *16S rRNA* gene is more suitable for *Demodex* detection and phylogenetic inference than the *18S rRNA* gene. Amplification of the 18S marker may lead to the unintended detection of non-target mites, such as soil mites, or, in some cases, to the failure to detect *Demodex* mites. The mitochondrial 16S marker, instead, showed greater sequence variability and, consequently, higher resolution at short evolutionary distances than the nuclear 18S marker. However, despite this increased variability, bootstrap support for the nodes separating the major *Demodex* lineages remained low, generally failing to reach the 0.9–0.95 threshold, except for the *folliculorum–injai* clade. A similar lack of strong support was observed in the 18S-based phylogeny. These results indicate that some uncertainty persists in the inferred relationships among *Demodex* species, highlighting the need for additional loci and increased sampling to robustly resolve species boundaries and evolutionary relationships within the genus.

### 4.2. Co-Infestations and Host Specificity Concerns

To date, no peer-reviewed studies have explicitly documented co-infestations of *D. canis* and *D. injai*, and *D. canis* and *D. brevis,* within canids. Most reports describe these species in isolation, as dogs exhibit clinical signs of demodicosis [1,17,58]. Similarly, studies conducted in healthy dogs have not reported co-infestations [13,21,22]. In our study, no known diseases were present in either population at the time, which supports previous findings that *Demodex* mites can be carried asymptomatically across various mammalian hosts [9,13,59]. The absence of confirmed co-occurrence in dogs may indicate underdiagnosis due to limitations in sampling strategies, as co-infestations of *D. canis* with *D. folliculorum* and *D. brevis* have been documented in two healthy cats [7]. Co-infestation of *D. brevis* and *D. folliculorum* has also been documented in humans, particularly in relation to dermatological and ophthalmological conditions, such as chronic blepharitis and rosacea [60,61,62]. These two species usually inhabit different ecological niches: *D. folliculorum* in hair follicles and *D. brevis* in sebaceous or meibomian glands, but they can coexist in the same individual [62], and their combined presence has been suggested as a potential factor in the development of these diseases [63,64].

The observation of interspecific co-infestations in some wolves, along with findings that the Iberian wolf harbors *Demodex* species found in dogs and humans, suggests substantial ecological plasticity and raises important questions about the strict host specificity paradigm. Previous studies have raised similar concerns by reporting the presence of *D. canis* not only in dogs and wolves, but also in a variety of hosts, including bats, ferrets, cats, and mice, and, exceptionally, in a single bear and a single human [7,9,11,31,65]. These findings support a revision of the host–parasite model for *Demodex*, proposing that at least some species, especially those infecting carnivores, may function as opportunistic generalists rather than obligate specialists. Molecular studies across a broader range of wild and domestic hosts are needed to further assess the extent of host specificity versus host plasticity in this group.

### 4.3. Inference on the Process of Dog Domestication

Contrary to our initial hypothesis, our results indicate that wolves and domestic dogs share closely related *Demodex* species, suggesting that long-term host divergence and domestication may not have led to the evolution of distinct mite species in these canids. The identification of *D. canis*, *D. injai*, *D. folliculorum*, and *D. brevis* in wild wolves is a remarkable finding that challenges the traditional view of strict host specificity in *Demodex* mites. While *D. canis* and *D. injai* are typically associated with domestic dogs, and *D. folliculorum* and *D. brevis* with humans, their concurrent presence in wolves raises several questions regarding their evolutionary history and host associations. The detection of *D. canis* and *D. injai* in Iberian wolves is not entirely surprising, whereas their absence from the skin of Italian wolves is unexpected. Further analysis of additional individuals would be necessary to confirm the presence of these mites in the Italian population. And even more surprising is the detection of human-associated *Demodex* in wolves. Two possible explanations can be proposed for this unexpected detection: 1, wolves served as a reservoir and potential source of transmission to humans, occurring through early interactions between canids and hominids during the process of dog domestication [2], and 2, a more plausible scenario involving ecological contact and host-switching events, given the recent isolation history of these two wolf populations. The Iberian and Italian wolf populations have been geographically isolated since the early 20th century, when wolves became extinct in France [52]. Since then, the population sizes of Spanish and Italian wolves have also declined dramatically, with minima reached in both countries during the 70s of the 20th century [51,52,66,67]. The presence of human-associated *Demodex* species in wolves may result from cross-species transmission, potentially facilitated by overlapping habitats, direct contact (e.g., in captivity or rehabilitation centers), or indirect contact through domestic dogs. However, to date, no reports have been published of dogs harboring human-associated *Demodex* on their skin. Future studies employing longer or more variable genetic markers alongside broader host and geographic sampling will be crucial to disentangle the evolutionary relationships of *Demodex* mites, clarify potential host-switching events, and enhance our understanding of their ecology and evolution in both wild and domestic hosts.

## Figures and Tables

**Table 1:** Number of positive (+) and negative (−) samples for *Demodex* analyzed by sex, wolf species, and region (ES: Spain; IT: Italy).

Region and Host	Female		Male		Total
	−	+	−	+	
Cantabria (ES)	40	20	40	26	126
Castilla y León (ES)	1				1
**Total** ***Canis lupus signatus***	41	20	40	26	127
Alessandria (IT)			1		1
Aragón (ES)			1		1
Catalunya (ES)				1	1
Cuneo (IT)	2	1	1	1	5
Torino (IT)	2	1		1	4
Vercelli (IT)				1	1
**Total** ***Canis lupus italicus***	4	2	3	4	13

**Table 2:** *Demodex* spp. detected (+) by site using the *18S rRNA* and *16S rRNA* genes with the corresponding GenBank accession number, *Demodex* variants identified, and the number of positive wolves using the *16S rRNA* gene.

*Demodex* sp.	N + Sites—18S	GenBanK—18S	N + Sites—16S	Variants—16S	GenBanK—16S	N + Sites—16S	N + Iberian Wolf—16S	N + Italian Wolf—16S
*D. brevis*	0	-	5	D.brevis_UABW1	PX232547	2	2	
				D.brevis_UABW2	PX232548	2	1	1 ^(5)^
				D.brevis_UABW3	PX232549	1	1 ^(3)^	
*D. folliculorum*	5	PX421031	12	D.folliculorum_UABW1	PX232550	7	1	4
				D.folliculorum_UABW2	PX232551	3		2 ^(5)^
				D.folliculorum_UABW3	PX232552	2	1	
*D. canis*	26	PX421029	30	D.canis_UABW1	PX232553	27	11 ^(1–4)^	
				D.canis_UABW2	PX232554	3	1	
*D. injai*	52	PX421030	54	D.injai_UABW1	PX232555	19	12 ^(1,4)^	
				D.injai_UABW2	PX232556	29	16 ^(2)^	
				D.injai_UABW3	PX232557	6	4	
Total	83		101			101	50	7

^(1–5)^ Co-infested wolves.

## Data Availability

All relevant data are within the paper. All new sequences are available from the GenBank database (accession numbers: PX232547, PX232548, PX232549, PX232550, PX232551, PX232552, PX232553, PX232554, PX232555, PX232556, PX232557, PX421029, PX421030, PX421031).

## Supplementary Materials

The following supporting information can be downloaded at: https://www.mdpi.com/article/10.3390/pathogens15030336/s1, Table S1: Sample collection.

## Abbreviations

The following abbreviations are used in this manuscript:

qPCR	Quantitative Polymerase Chain Reaction
Tm	Melting curve
Cp	Amplification cycle
SNP	Single Nucleotide Polymorphism
UAB	Universitat Autònoma de Barcelona
IUCN	International Union for Conservation of Nature
CITES	Convention on International Trade in Endangered Species of Wild Fauna and Flora

## References

[1] Ferrer L., Ravera I., Silbermayr K. (2014). Immunology and Pathogenesis of Canine Demodicosis. Vet. Dermatol..

[2] Thoemmes M.S., Fergus D.J., Urban J., Trautwein M., Dunn R.R. (2014). Ubiquity and Diversity of Human-Associated *Demodex* Mites. PLoS ONE.

[3] El-Moamly A., El-Swify O. (2025). Raising Awareness of *Demodex* Mites: A Neglected Cause of Skin Disease. Infection.

[4] Zhao Y.-E., Wu L.-P. (2012). Phylogenetic Relationships in *Demodex* Mites (Acari: Demodicidae) Based on Mitochondrial 16S rDNA Partial Sequences. Parasitol. Res..

[5] Izdebska J.N., Rolbiecki L. (2014). *Demodex lutrae* n. sp. (Acari) in European Otter *Lutra lutra* (Carnivora: Mustelidae) with Data from Other Demodecid Mites in Carnivores. J. Parasitol..

[6] Silbermayr K., Horvath-Ungerboeck C., Eigner B., Joachim A., Ferrer L. (2014). Phylogenetic Relationships and New Genetic Tools for the Detection and Discrimination of the Three Feline *Demodex* Mites. Parasitol. Res..

[7] Ferreira D., Sastre N., Ravera I., Altet L., Francino O., Bardagí M., Ferrer L. (2015). Identification of a Third Feline *Demodex* Species through Partial Sequencing of the 16S rDNA and Frequency of *Demodex* Species in 74 Cats Using a PCR Assay. Vet. Dermatol..

[8] Palopoli M.F., Fergus D.J., Minot S., Pei D.T., Simison W.B., Fernandez-Silva I., Thoemmes M.S., Dunn R.R., Trautwein M. (2015). Global Divergence of the Human Follicle Mite *Demodex folliculorum*: Persistent Associations between Host Ancestry and Mite Lineages. Proc. Natl. Acad. Sci. USA.

[9] Sastre N., Francino O., Curti J.N., Armenta T.C., Fraser D.L., Kelly R.M., Hunt E., Silbermayr K., Zewe C., Sánchez A. (2016). Detection, Prevalence and Phylogenetic Relationships of *Demodex* spp and Further Skin Prostigmata Mites (Acari, Arachnida) in Wild and Domestic Mammals. PLoS ONE.

[10] Nashat M.A., Luchins K.R., Lepherd M.L., Riedel E.R., Izdebska J.N., Lipman N.S. (2017). Characterization of *Demodex musculi* Infestation, Associated Comorbidities, and Topographic Distribution in a Mouse Strain with Defective Adaptive Immunity. Comp. Med..

[11] Esenkaya Taşbent F., Dik B. (2018). A dog related *Demodex* spp. infestation in a student: A rare *Demodex* case. Mikrobiyoloji Bülteni.

[12] Ilie M.S., Imre M., Giubega S., Luca I., Florea T., Morariu S. (2021). Feline Demodicosis Case Report—First Molecular Characterization of *Demodex* Mites in Romania. Pathogens.

[13] Ravera I., Altet L., Francino O., Sánchez A., Roldán W., Villanueva S., Bardagí M., Ferrer L. (2013). Small *Demodex* Populations Colonize Most Parts of the Skin of Healthy Dogs. Vet. Dermatol..

[14] Ricardo-Gonzalez R.R., Kotas M.E., O’Leary C.E., Singh K., Damsky W., Liao C., Arouge E., Tenvooren I., Marquez D.M., Schroeder A.W. (2022). Innate Type 2 Immunity Controls Hair Follicle Commensalism by *Demodex* Mites. Immunity.

[15] Crawford G.H., Pelle M.T., James W.D. (2004). Rosacea: I. Etiology, Pathogenesis, and Subtype Classification. J. Am. Acad. Dermatol..

[16] Chen W., Plewig G. (2014). Human Demodicosis: Revisit and a Proposed Classification. Br. J. Dermatol..

[17] Mueller R.S., Rosenkrantz W., Bensignor E., Karaś-Tęcza J., Paterson T., Shipstone M.A. (2020). Diagnosis and Treatment of Demodicosis in Dogs and Cats: Clinical Consensus Guidelines of the World Association for Veterinary Dermatology. Vet. Dermatol..

[18] Hill L.R., Kille P.S., Weiss D.A., Craig T.M., Coghlan L.G. (1999). *Demodex* Musculi in the Skin of Transgenic Mice. Contemp. Top. Lab. Anim. Sci..

[19] Gazi U., Taylan-Ozkan A., Mumcuoglu K.Y. (2019). Immune Mechanisms in Human and Canine Demodicosis: A Review. Parasite Immunol..

[20] Marquardt-Feszler A., Cekała K., Dębska-Ślizień A., Imko-Walczuk B. (2022). Demodicosis among Immunocompromised Patients: A Review. Postępy Dermatologii i Alergologii.

[21] Fondati A., De Lucia M., Furiani N., Monaco M., Ordeix L., Scarampella F. (2010). Prevalence of *Demodex* Canis-Positive Healthy Dogs at Trichoscopic Examination. Vet. Dermatol..

[22] Izdebska J.N., Fryderyk S. (2011). Diversity of Three Species of the Genus *Demodex* (Acari, Demodecidae) Parasitizing Dogs in Poland. Pol. J. Environ. Stud..

[23] Zhao Y.-E., Wu L.-P., Hu L., Xu Y., Wang Z.-H., Liu W.-Y. (2012). Sequencing for Complete rDNA Sequences (18S, ITS1, 5.8S, ITS2, and 28S rDNA) of *Demodex* and Phylogenetic Analysis of Acari Based on 18S and 28S rDNA. Parasitol. Res..

[24] Yabsley M.J., Clay S.E., Gibbs S.E.J., Cunningham M.W., Austel M.G. (2013). Morphologic and Molecular Characterization of a *Demodex* (Acari: Demodicidae) Species from White-Tailed Deer (*Odocoileus virginianus*). Int. Sch. Res. Not..

[25] Sallows T.A. (2007). Diet Preferences and Parasites of Grey Wolves in Riding Mountain National Park of Canada. Master of Environment Thesis.

[26] Stronen A.V., Sallows T.A., Forbes G.J., Wagner B., Paquet P.C. (2011). Diseases and Parasites in Wolves of the Riding Mountain National Park Region, Manitoba, Canada. J. Wildl. Dis..

[27] Cafiero S.A., Petroni L., Natucci L., Casale L., Raffaelli M., Baldacci D., Di Rosso A., Rossi C., Casulli A., Massolo A. (2025). Parasite Diversity in Grey Wolves (*Canis lupus*) from Tuscany, Central Italy: A Copromicroscopical Investigation. Int. J. Parasitol. Parasites Wildl..

[28] Zhou C.-F., Wu S., Martin T., Luo Z.-X. (2013). A Jurassic Mammaliaform and the Earliest Mammalian Evolutionary Adaptations. Nature.

[29] Murphy W.J., Eizirik E., O’Brien S.J., Madsen O., Scally M., Douady C.J., Teeling E., Ryder O.A., Stanhope M.J., de Jong W.W. (2001). Resolution of the Early Placental Mammal Radiation Using Bayesian Phylogenetics. Science.

[30] Frank L.A., Kania S.A., Chung K., Brahmbhatt R. (2013). A Molecular Technique for the Detection and Differentiation of *Demodex* Mites on Cats. Vet. Dermatol..

[31] Lankton J.S., Chapman A., Ramsay E.C., Kania S.A., Newkirk K.M. (2013). Preputial *Demodex* Species in Big Brown Bats (*Eptesicus fuscus*) in Eastern Tennessee. J. Zoo Wildl. Med..

[32] Palopoli M.F., Minot S., Pei D., Satterly A., Endrizzi J. (2014). Complete Mitochondrial Genomes of the Human Follicle Mites *Demodex brevis* and *D. folliculorum*: Novel Gene Arrangement, Truncated tRNA Genes, and Ancient Divergence between Species. BMC Genom..

[33] Brown S.K., Pedersen N.C., Jafarishorijeh S., Bannasch D.L., Ahrens K.D., Wu J.-T., Okon M., Sacks B.N. (2011). Phylogenetic Distinctiveness of Middle Eastern and Southeast Asian Village Dog Y Chromosomes Illuminates Dog Origins. PLoS ONE.

[34] Leonard J.A., Wayne R.K., Wheeler J., Valadez R., Guillén S., Vilà C. (2002). Ancient DNA Evidence for Old World Origin of New World Dogs. Science.

[35] Savolainen P., Zhang Y., Luo J., Lundeberg J., Leitner T. (2002). Genetic Evidence for an East Asian Origin of Domestic Dogs. Science.

[36] Axelsson E., Ratnakumar A., Arendt M.-L., Maqbool K., Webster M.T., Perloski M., Liberg O., Arnemo J.M., Hedhammar Å., Lindblad-Toh K. (2012). The Genomic Signature of Dog Domestication Reveals Adaptation to a Starch-Rich Diet. Nature.

[37] Germonpré M., Sablin M.V., Stevens R.E., Hedges R.E.M., Hofreiter M., Stiller M., Després V.R. (2009). Fossil Dogs and Wolves from Palaeolithic Sites in Belgium, the Ukraine and Russia: Osteometry, Ancient DNA and Stable Isotopes. J. Archaeol. Sci..

[38] Ovodov N.D., Crockford S.J., Kuzmin Y.V., Higham T.F.G., Hodgins G.W.L., van der Plicht J. (2011). A 33,000-Year-Old Incipient Dog from the Altai Mountains of Siberia: Evidence of the Earliest Domestication Disrupted by the Last Glacial Maximum. PLoS ONE.

[39] Davis S.J.M., Valla F.R. (1978). Evidence for Domestication of the Dog 12,000 Years Ago in the Natufian of Israel. Nature.

[40] vonHoldt B.M., Pollinger J.P., Lohmueller K.E., Han E., Parker H.G., Quignon P., Degenhardt J.D., Boyko A.R., Earl D.A., Auton A. (2010). Genome-Wide SNP and Haplotype Analyses Reveal a Rich History Underlying Dog Domestication. Nature.

[41] Skoglund P., Götherström A., Jakobsson M. (2011). Estimation of Population Divergence Times from Non-Overlapping Genomic Sequences: Examples from Dogs and Wolves. Mol. Biol. Evol..

[42] Wang G.-D., Zhai W., Yang H.-C., Wang L., Zhong L., Liu Y.-H., Fan R.-X., Yin T.-T., Zhu C.-L., Poyarkov A.D. (2015). Out of Southern East Asia: The Natural History of Domestic Dogs across the World. Cell Res..

[43] Bergström A., Stanton D.W.G., Taron U.H., Frantz L., Sinding M.-H.S., Ersmark E., Pfrengle S., Cassatt-Johnstone M., Lebrasseur O., Girdland-Flink L. (2022). Grey Wolf Genomic History Reveals a Dual Ancestry of Dogs. Nature.

[44] Freedman A.H., Gronau I., Schweizer R.M., Ortega-Del Vecchyo D., Han E., Silva P.M., Galaverni M., Fan Z., Marx P., Lorente-Galdos B. (2014). Genome Sequencing Highlights the Dynamic Early History of Dogs. PLoS Genet..

[45] Crandall K.A., Bininda-Emonds O.R., Mace G.M., Wayne R.K. (2000). Considering Evolutionary Processes in Conservation Biology. Trends Ecol. Evol..

[46] Carmichael L.E., Nagy J.A., Larter N.C., Strobeck C. (2001). Prey Specialization May Influence Patterns of Gene Flow in Wolves of the Canadian Northwest. Mol. Ecol..

[47] Geffen E., Anderson M.J., Wayne R.K. (2004). Climate and Habitat Barriers to Dispersal in the Highly Mobile Grey Wolf. Mol. Ecol..

[48] Musiani M., Leonard J.A., Cluff H.D., Gates C.C., Mariani S., Paquet P.C., Vilà C., Wayne R.K. (2007). Differentiation of Tundra/Taiga and Boreal Coniferous Forest Wolves: Genetics, Coat Colour and Association with Migratory Caribou. Mol. Ecol..

[49] Lucchini V., Galov A., Randi E. (2004). Evidence of Genetic Distinction and Long-Term Population Decline in Wolves (*Canis lupus*) in the Italian Apennines. Mol. Ecol..

[50] Pilot M., Branicki W., Jędrzejewski W., Goszczyński J., Jędrzejewska B., Dykyy I., Shkvyrya M., Tsingarska E. (2010). Phylogeographic History of Grey Wolves in Europe. BMC Evol. Biol..

[51] Sastre N., Vilà C., Salinas M., Bologov V.V., Urios V., Sánchez A., Francino O., Ramírez O. (2011). Signatures of Demographic Bottlenecks in European Wolf Populations. Conserv. Genet..

[52] Valiere N., Fumagalli L., Gielly L., Miquel C., Lequette B., Poulle M.L., Weber J.M., Arlettaz R., Taberlet P. (2003). Long-Distance Wolf Recolonization of France and Switzerland Inferred from Non-Invasive Genetic Sampling over a Period of 10 Years. Anim. Conserv..

[53] Librado P., Rozas J. (2009). DnaSP v5: A Software for Comprehensive Analysis of DNA Polymorphism Data. Bioinformatics.

[54] Posada D., Crandall K.A. (1998). MODELTEST: Testing the Model of DNA Substitution. Bioinformatics.

[55] Ronquist F., Teslenko M., van der Mark P., Ayres D.L., Darling A., Höhna S., Larget B., Liu L., Suchard M.A., Huelsenbeck J.P. (2012). MrBayes 3.2: Efficient Bayesian Phylogenetic Inference and Model Choice across a Large Model Space. Syst. Biol..

[56] Hillis D.M., Bull J.J. (1993). An Empirical Test of Bootstrapping as a Method for Assessing Confidence in Phylogenetic Analysis. Syst. Biol..

[57] Lorenzini R., Pizzarelli A., Attili L., Biagetti M., Sebastiani C., Ciucci P. (2026). Genetic Evidence Reveals Extensive Wolf-Dog Hybridisation in Peninsular Italy: Warnings against Ineffective Management. Biol. Conserv..

[58] Saridomichelakis M., Koutinas A., Papadogiannakis E., Papazachariadou M., Liapi M., Trakas D. (1999). Adult-Onset Demodicosis in Two Dogs Due to *Demodex* Canis and a Short-Tailed Demodectic Mite. J. Small Anim. Pract..

[59] Fryderyk S., Izdebska J.N. (2001). *Demodex* Phylloides (Acari, Demodecidae) as a Specific Parasite of *Sus scrofa* (Mammalia, Artiodactyla). Wiadomości Parazytologiczne.

[60] Biernat M.M., Rusiecka-Ziółkowska J., Piątkowska E., Helemejko I., Biernat P., Gościniak G. (2018). Occurrence of *Demodex* Species in Patients with Blepharitis and in Healthy Individuals: A 10-Year Observational Study. Jpn. J. Ophthalmol..

[61] Pyzia J., Mańkowska K., Czepita M., Kot K., Łanocha-Arendarczyk N., Czepita D., Kosik-Bogacka D.I. (2023). *Demodex* Species and Culturable Microorganism Co-Infestations in Patients with Blepharitis. Life.

[62] Kosik-Bogacka D., Łanocha-Arendarczyk N., Pilarczyk R., Schneider-Matyka D., Kot K., Grzeszczak K., Pyzia J., Grochans E. (2025). Ocular Symptoms in Pre- and Perimenopausal Woman Infected with *Demodex* spp.. Pathogens.

[63] Fromstein S.R., Harthan J.S., Patel J., Opitz D.L. (2018). *Demodex* Blepharitis: Clinical Perspectives. Clin. Optom..

[64] Zeytun E., Yazici M. (2024). Human *Demodex* Mites (Acari: Demodicidae) as a Possible Etiological Factor in Rosacea: A Cross-Sectional Study from Turkey. Syst. Appl. Acarol..

[65] Oleaga Á., Fayos M., Balseiro A., Borragán S., de Pedro G., Armenteros J.Á., Balsera R., Moreiro M., Sastre N., Ferrer L. (2024). Demodicosis in a Free-Ranging Eurasian Brown Bear (*Ursus arctos arctos*) Cub in the Endangered Cantabrian Population, Spain. J. Wildl. Dis..

[66] Valverde J. (1971). El Lobo Español. Montes.

[67] Zimen E., Boitani L. (1974). Number and Distribution of Wolves in Italy. Zeitschrift für Säugetierkunde.

